# Neurenteric Cysts Found on the Outer Membrane of a Subdural Haematoma

**DOI:** 10.1155/2013/130597

**Published:** 2013-09-10

**Authors:** Satoshi Takahashi, Yoshio Tanizaki, Kazunori Akaji, Tadashige Kano, Ban Mihara, Nobuhide Masawa

**Affiliations:** ^1^Department of Neurosurgery, Institute of Brain and Blood Vessels, Mihara Memorial Hospital, Ota-machi 366, Isesaki, Gunma 372-0006, Japan; ^2^Department of Neurology, Institute of Brain and Blood Vessels, Mihara Memorial Hospital, Ota-machi 366, Isesaki, Gunma 372-0006, Japan; ^3^Department of Anatomic and Diagnostic Pathology, Dokkyo University School of Medicine, Kitakobayashi 880, Mibu, Shimotsuga, Tochigi 321-0293, Japan

## Abstract

We report on a patient initially diagnosed with a chronic subdural haematoma that was resistant to treatment. After the second burr hole craniostomy within a half month failed to resolve the subdural haematoma (SDH), we performed a craniotomy to identify the point of bleeding. Macroscopic evaluation showed that most of the outer membrane of the SDH was transparent; however, further examination revealed the presence of multiple white regions. Pathologic examination showed that the white regions were fluid filled and surrounded by columnar ciliated epithelial cells. These lesions were pathologically diagnosed as neurenteric cysts. To our knowledge, this is the first report on a patient with neurenteric cysts found on the outer membrane of a CSDH. We agree that a craniotomy is a treatment of last resort for recurrent CSDHs; however, sometimes this procedure can be very useful for identifying underlying causes of obstinate SDHs as well as for their treatment.

## 1. Introduction

Neurenteric cysts are rare congenital cystic lesions that originate from displaced elements of the alimentary canal. They account for 0.01% of central nervous system (CNS) tumours and are more frequently found in the cervicothoracic region with an intradural, extramedullary location [1, 2]. Intracranial neurenteric cysts are very rare, and the majority are located in the posterior fossa. Even more infrequent are neurenteric cysts that arise supratentorially [1].

In this report, we describe a patient with neurenteric cysts found on the outer membrane of a chronic subdural haematoma (CSDH). Thus far, 2 patients who have presented with spontaneous haemorrhaging into a neurenteric cyst have been reported [3, 4]; however, this is the first report of a patient with neurenteric cysts found on the outer membrane of a CSDH. 

## 2. Case Presentation

### 2.1. Onset

**Figure 1:**
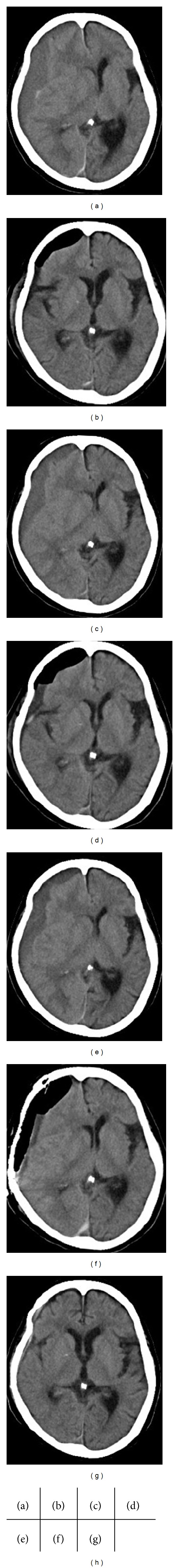
CT images. (a) Before the first drainage; (b) 1 day after the first drainage; (c) before the second drainage; (d) 1 day after the second surgery; (e) before the third surgery (i.e., craniotomy); (f) after craniotomy; (g) 2.5 months after craniotomy.

An 80-year-old woman with no prior history of neurological symptoms was referred to our hospital under a diagnosis of CSDH. On admission, the patient was disoriented, had headache, and presented gait disturbance. Additionally, the patient's family documented progressive memory disturbance over the course of the last several days. CT revealed a CSDH over the right convexity and a 15 mm midline shift (Figure 1(a)).

### 2.2. Initial Treatment

In order to prevent brain herniation, emergent burr hole craniostomy (BHC) for the CSDH was performed under local anaesthesia. A second CT scan performed 1 day after the operation revealed that most of the haematoma was well drained, and the midline shift was relieved (Figure 1(b)). Additionally, the patient's symptoms of headache, gait disturbance, and disorientation were alleviated, and the patient was discharged from the hospital 7 days after drainage with no apparent sign of subdural haematoma (SDH) recurrence.

### 2.3. Second BHC

Six days after discharge, the patient presented again with gait disturbance, and CT revealed SDH recurrence (Figure 1(c)). We performed another BHC on the patient (by using the same burr hole), and an additional CT scan was performed 1 day after the second drainage (Figure 1(d)); it revealed almost complete haematoma removal. Following the second surgery, the patient's symptoms (including gait disturbance) were again relieved, albeit, temporarily. Later, along with recurrence and progression of symptoms, the volume of the haematoma was found to be increasing, as confirmed by follow-up CT (Figure 1(e)).

### 2.4. Craniotomy

**Figure 2:**
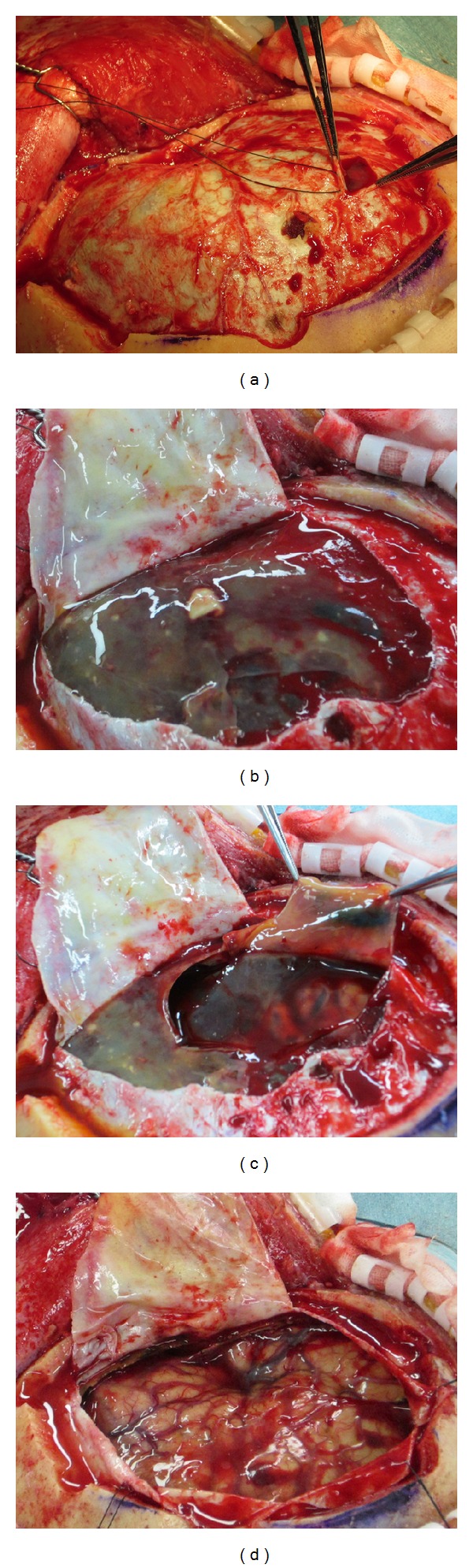
Intraoperative pictures. (a) After craniotomy; (b) after dural opening, the outer membrane of the subdural haematoma was transparent and jelly like and contained multiple white regions; (c) after haematoma removal; (d) absence of abnormalities on the brain surface following haematoma removal.

**Figure 3:**
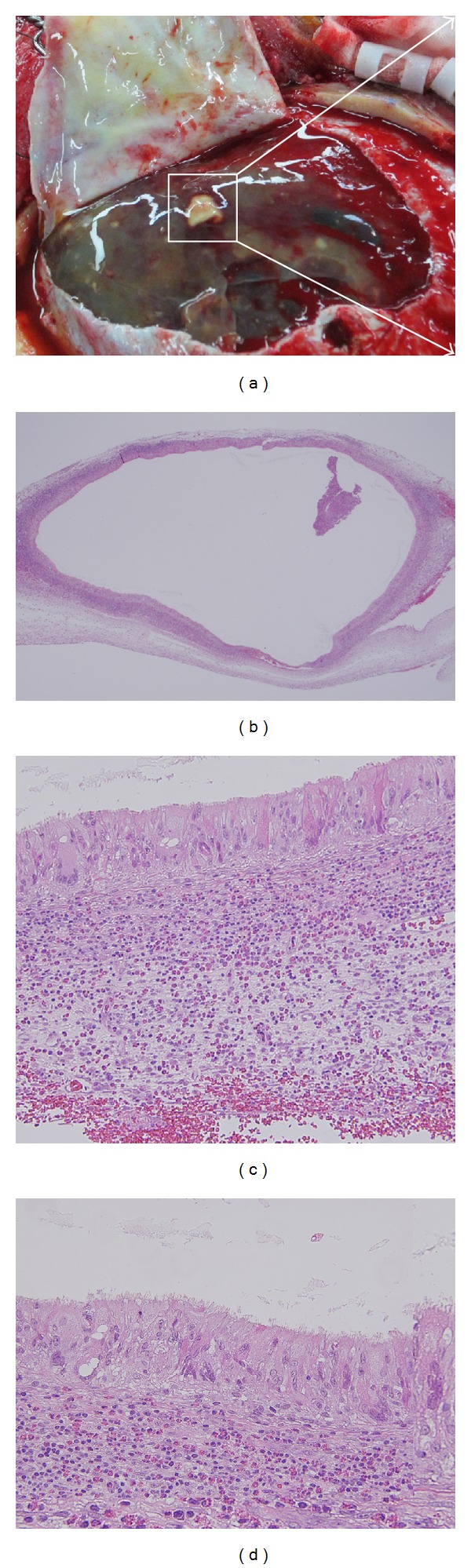
Pathological findings. (a) After dural opening, the outer membrane of the subdural haematoma was transparent and jelly like and contained multiple white regions; (b) 2x HE staining; (c) 20x HE staining; (d) 40x HE staining.

After two BHCs within 2 weeks failed to resolve the patient's SDH, we decided to perform a craniotomy in order to rule out any complications that might have affected the SDH. Under general anaesthesia, a skin incision was made behind the hairline at midline, and then extended in a “question mark” shape, terminating in front of the ear, close to the level of the zygoma. A series of burr holes was then made in the skull (it was not necessary to extensively drill the sphenoid ridge), and the skull was cut between two adjacent burr holes in a progressive manner until a bone flap could be separated from the surrounding skull. The bone flap was then removed to reveal the frontotemporal region (Figure 2(a)), and the dura was opened in a curvilinear fashion with the base in the frontobasal direction (Figure 2(b)). The outer membrane of the SDH was revealed to be transparent and contained multiple white regions (Figures 2(b) and 3(a)). The SDH was then evacuated and washed out using artificial cerebrospinal fluid (CSF) (Figure 2(c)). The inner membrane of the haematoma cavity was also removed since it did not tightly adhere to the arachnoid membrane, and since we observed fluid not only between the membranes, but also under the inner membrane. After evacuation of the haematoma, the right frontotemporal brain region was exposed, and we could not find any further abnormalities on the surface of the brain (Figure 2(d)). 

### 2.5. Pathological Findings

The inner and outer membranes of the haematoma, including the white regions, were removed and sent for analysis. Pathologic examination revealed that the contents of the white regions were fluid filled and surrounded by columnar ciliated epithelial cells (Figures 3(b)–3(d)). Therefore, the lesions were pathologically diagnosed as neurenteric cysts.

### 2.6. Postoperative Course after Craniotomy

A CT scan performed immediately after the craniotomy confirmed almost complete removal of the haematoma (Figure 1(f)). Another CT scan performed 1 day after the operation showed a subdural fluid collection that required a second drainage. After that, the right side of the subdural space gradually disappeared. The patient was discharged from the hospital 1 month after the craniotomy without any neurological deficit. A follow-up CT scan performed 2.5 months after the craniotomy showed no apparent sign of SDH recurrence (Figure 1(g)).

## 3. Discussion

CSDH is a common disease especially in the elder people. Three principal techniques have been reported to evacuate haematomas, including BHC, twist-drill craniostomy, and craniotomy [5]. Compared to craniotomies, burr hole and twist drill craniotomies are considered safer surgical procedures. In fact, BHC is preferred over craniotomy even in cases of recurrent haematoma [5]. Consequently, craniotomy is considered the last choice in the treatment for recurrent CSDH, and therefore, we routinely use BHC as a surgical strategy for CSDH. In the current case, a single burr hole was made under local anaesthesia. After the dura was opened, the outer membrane of the CSDH was cut and a silicon tube was inserted into the haematoma cavity. The silicon tube was then connected to a closed drainage system. In other case studies, irrigation with artificial CSF via a drainage tube has been performed [6]. From our experience, 90% of patients make a full recovery after this treatment (unpublished data). In cases where CSDH recurs, we typically perform a BHC again by using either the same burr hole or a new burr hole. Theoretically, 10% of patients will have one recurrence of CSDH, and of this, 10% will have another emergence of CSDH (i.e., 1% of all the patients). It is at this latter stage that we speculate that some specific underlying problems might have an effect on the SDH of the patient, and we then conduct a craniotomy. In the present case, CSDH of the patient recurred twice within a 2-week period; hence, we decided that performing a craniotomy was the correct course of treatment. 

In the present case, the SDH was resistant to treatment. Obstinate SDHs often have some specific underlying causes that lead to recurrence. Patients on anticoagulation therapy such as warfarin [7], patients with coagulopathy [8], or patients with CNS tumours [9] have been known to have persistent SDHs. In the present case, the SDH of the patient consistently increased until the patient underwent a craniotomy with extensive removal of the haematoma membrane that contained neurenteric cysts. This suggests that the neurenteric cysts found on the outer membrane of the SDH were one of the main reasons behind recurrence. While SDHs associated with CNS tumours such as dural metastasis [10] or subdural rhabdomyosarcoma [9] have been reported, there has so far been no reports on a SDH associated with a neurenteric cyst.

Neurenteric cysts are rare congenital clinical entities originating from displaced elements of the alimentary canal [1, 2]. They are more frequently found in the cervicothoracic region with an intradural, extramedullary location [1, 2]. Intracranial neurenteric cysts are rare, and in cases where they have been reported, they are typically located in the posterior fossa [1, 2]. To our knowledge, only three patients have presented with lateral extradural, supratentorial neurenteric cysts so far, [11–13] and none of the previously reported cases were associated with an SDH.

In the present case, the white regions found after the dura was opened contained a white fluid that was surrounded by columnar ciliated epithelial cells. On the basis of this pathological finding, we think that the SDH cavity itself was not a neurenteric cyst, but that multiple neurenteric cysts that existed on the outer membrane of the SDH were. Consistent with this finding, Kimura et al. reported a case of a patient with an intracranial neurenteric cyst with extensive dissemination [14]. Dissemination is one possible reason for the multiple neurenteric cysts found in the present case.

Finally, only two cases of spontaneous haemorrhage into neurenteric cysts have been previously reported [3, 4]. In the present case, the size of the SDH did not stop increasing until the patient underwent craniotomy with extensive removal of the haematoma membrane that contained multiple neurenteric cysts. Taken together, spontaneous haemorrhaging from neurenteric cysts into SDH cavities might account for the frequent recurrence of SDH in the current patient and in other patients as well.
